# Supplementation of Rosemary Extract Improves Lactation Performance and Rumen Function in Dairy Buffaloes Under Hot Weather

**DOI:** 10.3390/ani16020216

**Published:** 2026-01-11

**Authors:** Yinghui Li, Chenglong Li, Hanxing Yao, Pingting Liu, Mengwei Li, Xingguo Huang, Chengjian Yang

**Affiliations:** 1 Guangxi Key Laboratory of Buffalo Genetics, Reproduction and Breeding, Guangxi Buffalo Research Institute, Nanning 530001, China; liyinghui16@163.com (Y.L.);; 2 College of Animal Science and Technology, Hunan Agricultural University, Changsha 410128, China; 3 Yuelushan Laboratory, Changsha 410128, China

**Keywords:** dairy buffalo, heat stress, lactation performance, rosemary extract, rumen microbes, ruminal fermentation

## Abstract

Rosemary extract (RE), rich in carnosic and rosmarinic acid, which have antibacterial/antioxidant/anti-inflammatory effects, is a potential natural feed supplement for heat-stressed dairy buffaloes. This study systematically evaluated dietary RE effects on dairy buffaloes during hot weather. Twenty *Mediterranean* dairy buffaloes were randomly allocated into two groups for a 35-day trial: the control (CON) group receiving a basal diet, and the RE group receiving the same basal diet supplemented with 20 g/d of RE. Results showed RE tended to reduce buffalo body surface temperature; increased milk production, 4% fat-corrected milk, milk protein, lactose, and solids-not-fat; and optimized milk fatty acid profiles. In regard to blood, the RE group exhibited higher catalase activity, total antioxidant capacity, and concentrations of immunoglobulin A and M, together with lower concentrations of the pro-inflammatory cytokines interleukin-1β and tumor necrosis factor-α. Additionally, RE markedly elevated concentrations of total volatile fatty acid, acetate, propionate, and butyrate; improved microbial α-diversity indices (Sobs and Ace); and increased the abundances of *Rikenellaceae_RC9_gut_group* and *Butyrivibrio* spp., as well as the enrichment of multiple genera belonging to the family *Lachnospiraceae*. In conclusion, supplementing the diet of heat-stressed dairy buffaloes with 20 g/d of RE improves milk production and composition-related performance by optimizing the rumen ecosystem and enhancing systemic health status, with these effects observed as short-term responses under the conditions of the present study.

## Simple Summary

In the hot and humid summer, dairy buffaloes are prone to heat stress, characterized by reduced lactation performance, impaired immune function, and ruminal microbial imbalance, severely restricting the efficient and sustainable development of the dairy buffalo breeding industry. Rosemary extract (RE), a natural substance with antibacterial, antioxidative, and anti-inflammatory activities, has been extensively applied in cosmetics, food, and feed manufacturing. However, whether RE regulates heat-stressed buffaloes remains unclear. The experiment aimed to evaluate the effect of dietary RE supplementation on the lactation performance and rumen function of dairy buffaloes in hot weather. Results showed that RE supplementation slightly reduced the body surface temperature of buffaloes. More importantly, it increased milk production and beneficial components, such as milk protein and lactose, and optimized the milk fatty acid profile. In terms of health, RE enhanced the antioxidant capacity and immunity function of buffaloes and reduced inflammatory substances in the blood. It also enhanced rumen fermentation capacity and elevated the diversity of beneficial ruminal microorganisms. This study highlights RE’s potential as a natural feed supplement to boost the productivity of heat-stressed dairy buffaloes over the short term by optimizing the rumen environment and enhancing body health, providing practical value for hot-climate dairy buffalo farming.

## 1. Introduction

The escalating frequency and intensity of heat waves driven by global climate change have become critical challenges to livestock sustainability, especially in tropical and subtropical areas where environmental temperatures go beyond the thermoneutral range of ruminants [1,2]. Among ruminant species, water buffaloes (*Bubalus bubalis*) are especially vulnerable to heat stress owing to inherent physiological limitations: the sparse sweat gland distribution, dense epidermal layers, and melanin-rich pelage collectively impair thermoregulatory efficiency by hindering evaporative and radiative heat loss [3,4]. Chronic heat exposure triggers a cascade of pathological responses, including hyperthermia, oxidative stress-mediated cellular damage, and immune suppression, which ultimately compromise lactation productivity [5,6].

To mitigate these adverse effects, bioactive dietary interventions have emerged as practical and sustainable strategies. Rosemary (*Rosmarinus officinalis* L.), a perennial aromatic herb of the *Lamiaceae* family, is rich in bioactive constituents like carnosic acid, carnosol, and rosmarinic acid, properties that have fueled its widespread use in the cosmetics, food, and pharmaceutical industries [7]. Beyond these fields, rosemary’s antibacterial, antioxidative, and anti-inflammatory properties have extended its application to livestock production [8,9]. For monogastric animals, extensive research has confirmed that dietary rosemary extract (RE) supplementation improves growth traits, enhances antioxidative capacity, regulates gut microbial composition, and modulates humoral immune function [10,11,12,13]. In ruminants, the antioxidative activity of rosemary was first confirmed via the administration of an acute dose to sheep through a duodenal T-cannula. Nevertheless, the unique rumen ecosystem of ruminants, characterized by complex microbial fermentation, may alter the bioavailability and efficacy of RE, thereby influencing its final biological effects. Subsequent feeding trials have demonstrated that incorporating rosemary or its by-products into ruminant diets improves lactation performance in dairy sheep, goats, and cows [14,15,16]. This is particularly relevant for dairy ruminants, which are inherently prone to oxidative stress due to high metabolic demands (e.g., lactation) and environmental factors like heat stress; factors that not only impair rumen function but also disrupt milk production [17]. Moreover, multi-omics studies have further highlighted that the ruminal microbial community structure and function profiles are tightly coupled to lactation performance and overall health in dairy ruminants [18,19]. While RE has shown promise in modulating rumen microbiota and enhancing antioxidant status in various dairy animals, its effects on dairy buffaloes, especially under heat stress conditions, remain understudied.

Therefore, this research was designed to explore the impacts of supplementing RE in the diet on milk production performance, antioxidative capacity, immune function, rumen fermentation profiles, and ruminal microbial composition of dairy buffaloes in hot environments. By addressing this key research gap, the findings may provide evidence-based recommendations for optimizing dairy buffalo production and resilience in challenging environmental contexts.

## 2. Materials and Methods

### 2.1. Animal Ethics

All experimental protocols involving animals were endorsed by the Animal Ethics and Welfare Committee of the Guangxi Buffalo Research Institute (Nanning, China) under the authorization No. 20240801L.

### 2.2. Rosemary Extract

The RE was supplied by Hunan Zhizhiyuan Biotechnology Co., Ltd. (Luxi, China), which was prepared via solvent extraction from locally sourced rosemary leaves. The extraction method of RE and the detection protocol for its functional components (carnosic acid and rosmarinic acid) were performed according to the method described by Kong et al. [16]. The concentrations of carnosic acid and rosmarinic acid in the RE were determined to be 42.28 g/kg and 1.59 g/kg, respectively.

### 2.3. Animals and Experimental Design

The experiment was carried out from August to September at the Guangxi Buffalo Research Institute, situated in Nanning, South China (22°53′22.59″ N, 108°21′51.19″ E). Twenty *Mediterranean* dairy buffaloes with an average BW of 605 ± 22 kg, parity of 2.63 ± 1.26, and lactation stage of 118 ± 19 d were selected and assigned to two dietary treatments using a completely randomized design stratified by pre-experimental milk production and parity: a control group (CON) receiving a basal diet, and a RE-supplemented group (RE) fed the basal diet supplemented with 20 g/d of RE per buffalo. Daily top-dressing of RE was conducted by mixing it with a small portion of the total mixed ration (TMR) during morning feeding (prior to milking), and each experimental buffalo was observed continuously after feeding to ensure 100% consumption of the supplement. Daily feed orts of individual buffaloes were recorded to monitor feed intake. The experiment spanned 35 days, with a 7-day adaptation period included. Detailed feeding and housing management practices were consistent with those reported in our previous study [20]. The chemical composition of the basal diet is shown in Table 1. Individual dry matter intake (DMI) was computed daily by weighing the provided feed and orts per animal.

### 2.4. Assessment of Thermal Comfort Status

The physiological index (P3) model developed by Li et al. [21], validated for evaluating the thermal status of buffaloes in hot and humid environments, was adopted to assess their thermal comfort. The P3 index is computed using the formula: P3 = 0.654 × BST (°C) + 0.381 × RR (breaths/min). Based on the P3 values, thermal comfort is classified into four grades: comfort (P3 ≤ 25.30), danger (25.30 < P3 ≤ 28.64), stress (28.64 < P3 < 31.98), and emergency (P3 ≥ 31.98).

### 2.5. Recording of Physiological Parameters

Physiological parameters were measured twice weekly (Monday and Thursday) in the morning (08:00–09:00) and afternoon (14:30–15:30). Respiratory rate (RR, breaths/min) was manually counted: chest and abdominal movements were observed for 2 min with a stopwatch, and the average movements per minute were recorded. Body surface temperature (BST, °C) was measured using an infrared thermometer (HRQ-S60, Haorunqi Electronic Technology Co., Ltd., Zhengzhou, Henan, China), with the average temperature of the forehead, left chest, and left abdomen taken as the BST value. Rectal temperature (RT, °C) was assessed via a rectal thermometer (GLA 700, GLA Co., San Luis Obispo, CA, USA), which was retained in the rectum for 2 min to record the stabilized maximum temperature.

### 2.6. Feed and Fecal Samples Collection and Analysis

Feed samples were collected once weekly throughout the experimental period and immediately stored at −20 °C. Fecal samples were collected during the final 3 consecutive days of the experimental period. Fresh feces were weighed and homogenized daily, after which 10 mL of 10% (*v*/*v*) sulfuric acid (H_2_SO_4_) was added to each daily composite fecal sample to fix nitrogen; the samples were then stored at −20 °C for subsequent analysis. Subsequently, the frozen samples were thawed, dried in a forced-air oven at 65 °C to a constant weight, and ground to pass through a 40-mesh sieve. The nutrient composition, including crude protein (CP), neutral detergent fiber (NDF), acid detergent fiber (ADF), and ether extract (EE), was analyzed following the procedures described by the Association of Official Analytical Chemists (AOAC, 2005) [22], and gross energy (GE) was measured using an oxygen bomb calorimeter (SDACM3100, Hunan Sande Technology Co., Ltd., Xiangtan, China). The apparent total tract digestibility of each nutrient was calculated using acid-insoluble ash as an internal marker.

### 2.7. Milk Sample Collection and Analysis

Buffaloes were milked twice daily (05:00 and 14:00), with milk production recorded at each session. Weekly milk samples were collected for composition analysis, including milk fat, protein, lactose, solids-not-fat (SNF), total solids (TS), milk urea nitrogen (MUN), and free fatty acids (FFA), using an automatic milk composition analyzer (MilkoScan F120, FOSS, Hillerød, Denmark). The 4% fat-corrected milk (4% FCM) was computed via the following formula: 4% FCM = 0.4 × milk production (kg/d) + 0.15 × milk fat percentage (%) × milk production (kg/d). Milk samples for medium- and long-chain fatty acid profile analysis were collected and analyzed via GC-MS following our recently described method [20], with a total of 38 primary fatty acids quantified, including 15 saturated fatty acids (SFA), 10 monounsaturated fatty acids (MUFA), and 13 polyunsaturated fatty acids (PUFA).

### 2.8. Blood Samples Collection and Analysis

On the final day of the experiment, prior to morning feeding, blood samples (approximately 20 mL) were collected via the coccygeal vein of the buffaloes using the disposable vacuum blood collection tubes (non-anticoagulant tubes), and centrifuged to separate serum and plasma, respectively. Serum levels of total protein (TP), albumin (ALB), alkaline phosphatase (ALP), creatine kinase (CK), lactate dehydrogenase (LDH), aspartate aminotransferase (AST), alanine transaminase (ALT), urea nitrogen (UN), glucose (GLU), triglyceride (TG), total cholesterol (TC), immunoglobulin A (IgA), IgG, IgM, triiodothyronine (T3), tetraiodothyronine (T4), and insulin (INS) were quantified using an automatic blood biochemical analyzer (Cobas c311, Roche, Switzerland) with matching biochemical Kits. For plasma samples, the activities of catalase (CAT), glutathione peroxidase (GSH-PX), and superoxide dismutase (SOD), as well as total antioxidant capacity (T-AOC) and malondialdehyde (MDA) concentrations, were assayed using commercial kits purchased from Nanjing Jiancheng Bioengineering Institute (Nanjing, China). Additionally, serum concentrations of heat shock protein 70 (HSP70), serum amyloid A (SAA), interleukin-1beta (IL-1β), IL-2, IL-6, tumor necrosis factor-α (TNF-α), and interferon-γ (IFN-γ) were detected via ELISA kits (Jiangsu Jingmei Biotechnology, Taixing, China) following the manufacturers’ protocols.

### 2.9. Ruminal Contents Collection and Analysis

On the final day of the experiment, prior to morning feeding, approximately 500 mL of ruminal contents was collected from each buffalo, strained through two layers of cheesecloth, and subsamples for the analysis of ruminal fluid pH, microbial crude protein (MCP), ammonia nitrogen (NH3-N), and volatile fatty acids (VFA) were stored at −20 °C and determined as previously described [20]. Subsamples for DNA extraction were stored at −80 °C. Total genomic DNA was extracted from rumen fluid using an E.Z.N.A Soil DNA Kit (Omega Bio-Tek, Norcross, GA, USA), and subsequently, high-throughput Illumina MiSeq sequencing of the 16S rRNA gene V3–V4 region was performed as reported in our previous work [20]. α-diversity indices (Chao 1, Ace, Shannon, Simpson) were calculated using QIIME 2. β-diversity was analyzed through Bray–Curtis distance matrices, principal coordinate analysis (PCoA), and analysis of similarities (ANOSIM). Linear discriminant analysis (LDA) effect size (LEfSe) was used to identify significantly differential taxa (LDA score > 2.5, *p* < 0.05).

### 2.10. Statistical Analysis

All data were analyzed using the independent samples *t*-test in SPSS version 23 (IBM Corp., Armonk, NY, USA). Prior to statistical analysis, the normality of data distribution was verified using the Shapiro–Wilk test, and the homogeneity of variance was assessed via Levene’s test. For comparisons of microbial composition and α-diversity indices, Welch’s *t*-test was employed due to the presence of heterogeneous variances across groups. Results are presented as means ± standard error of the mean (SEM). Statistical significance was considered at *p* < 0.05, and a tendency was defined as 0.05 ≤ *p* < 0.10.

## 3. Results

### 3.1. Thermal Comfort State

**Figure 1:**
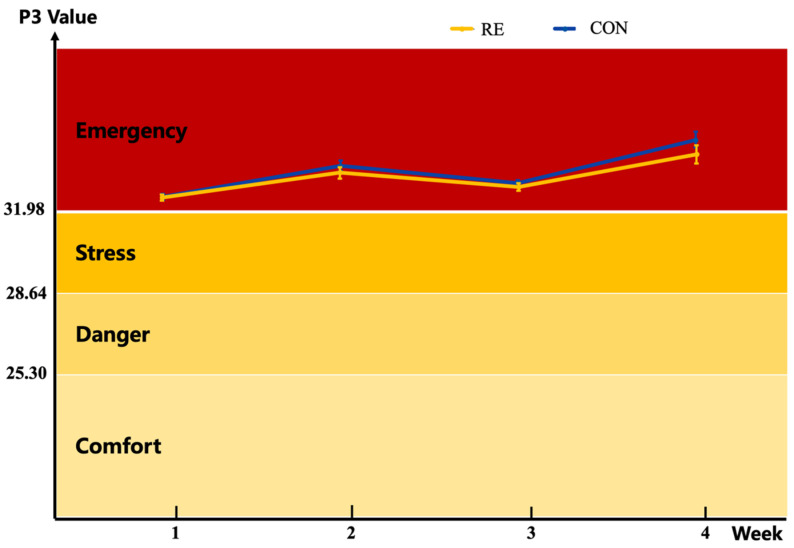
Physiological index model of *Mediterranean* dairy buffaloes during the experiment. The buffalo physiological index variable can be derived from this index model (practical model) as follows: P3 = 0.654 BST (°C) + 0.381 RR (breaths/min). Buffalo status categories: comfort (P3 ≤ 25.30), danger (25.30 < P3 ≤ 28.64), stress (28.64 < P3 < 31.98), and emergency (P3 ≥ 31.98).

During the experiment, P3 values of both groups exceeded the “emergency” threshold (P3 ≥ 31.98, Figure 1), indicating that all buffaloes experienced severe and persistent heat stress. Notably, P3 values of the RE group were consistently lower than those of the CON group.

### 3.2. Physiological Indices

As shown in Table 2, RE supplementation tended to reduce BST (*p* = 0.073) and decreased RR by 7.38% (not significant) compared with the CON group. No significant difference in RT was observed between groups (*p* > 0.05).

### 3.3. Feed Intake and Nutrient Apparent Digestibility

Results revealed no significant effect of RE supplementation on DMI or the nutrient apparent digestibility parameters, including CP, NDF, ADF, and EE digestibility (*p* > 0.05; Table 3).

### 3.4. Milk Production and Composition

Compared with the CON group, RE supplementation increased milk production, 4% FCM, and the percentage of milk protein, lactose, and SNF (*p* < 0.05; Table 4). It also tended to increase milk FFA content (*p* = 0.057). No significant differences in milk fat, TS, or MUN were observed between groups (*p* > 0.05).

### 3.5. Milk Fatty Acid Composition

As presented in Table 5 and Table S1, in comparison to the CON group, the RE group had lower concentrations of major SFA (C14:0, C18:0; *p* < 0.05), as well as higher levels of certain MUFAs, such as C20:1 (*p* < 0.05) and C22:1n9 (*p* = 0.074), and PUFAs (C20:3n3, C22:5n3, C22:5n6; *p* < 0.05). As a result, RE supplementation tended to decrease total SFA content (*p* = 0.085) and the SFA/UFA ratio (*p* = 0.061), while leaning towards increasing total PUFA content (*p* = 0.095).

### 3.6. Blood Biochemical, Hormonal, Antioxidative, and Immune Indices

Relative to the CON diet, RE supplementation showed a tendency to elevate serum ALP concentration (*p* = 0.055) while reducing serum TC and T4 concentrations (*p* = 0.075 and 0.067, respectively; Table 6). No significant difference in other serum biochemical indices or hormone levels was observed (*p* > 0.05). Regarding antioxidant status, the RE group exhibited significantly higher plasma CAT activity and T-AOC concentration (*p* < 0.05), whereas no significant differences were observed in GSH-PX, SOD, or MDA levels (*p* > 0.05). For immune indices, RE supplementation significantly increased serum IgA and IgM concentrations (*p* < 0.05), showed a tendency to elevate serum HSP70 concentration (*p* = 0.073), and notably reduced serum IL-1β and TNF-α concentrations (*p* < 0.05).

### 3.7. Ruminal Fermentation Profile

RE supplementation significantly increased the concentrations of TVFA, acetate, propionate, and butyrate in ruminal fluid (*p* < 0.001; Table 7). No significant differences in ruminal pH, MCP, NH3-N, isobutyrate, valerate, isovalerate, or the A:P ratio were observed between groups (*p* > 0.05).

### 3.8. Taxonomic Configuration of Ruminal Bacteria

**Figure 2:**
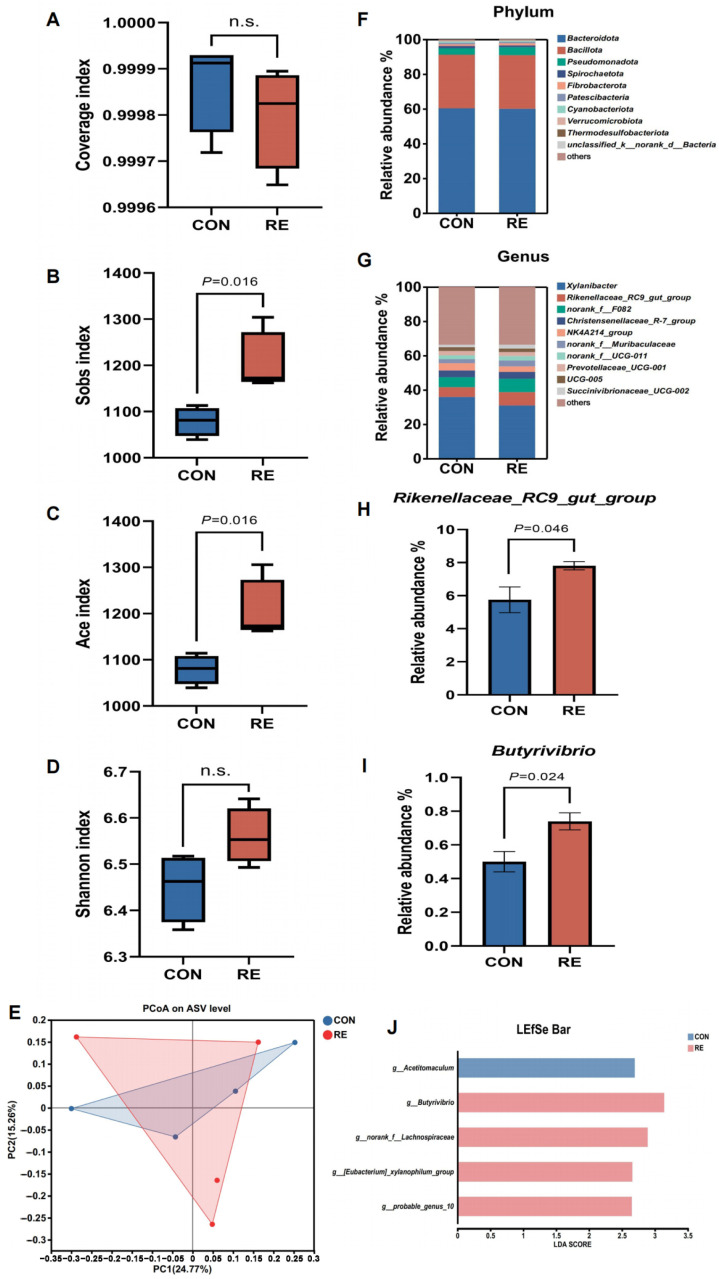
Effects of rosemary extract (RE) supplementation on diversity and composition of the rumen bacterial community in *Mediterranean* dairy buffaloes under hot weather. (**A**) Coverage index. (**B**) Sobs index. (**C**) Ace index. (**D**) Shannon index. (**E**) Beta diversity based on the principal coordinate analysis (PCoA) using the distance matrices generated from the Bray–Curtis analysis. (**F**) Relative abundance of the bacteria community at the phylum level. (**G**) Relative abundance of the bacteria community at the genus level. (**H**) Relative abundance of *Rikenellaceae_RC9_gut_group* genus. (**I**) Relative abundance of the *Butyrivibrio* genus. (**J**) Linear discriminant analysis effect size approach identifying biomarker genera between two groups. n.s., not significant.

No significant difference in the coverage index of ruminal microbiota was observed between groups, indicating consistent sequencing depth (Figure 2A). Relative to the CON group, the RE group exhibited higher α-diversity indices (Sobs, Ace; *p* < 0.05), whereas the Shannon index showed no significant variation (*p* > 0.05; Figure 2B–D). PCoA of β-diversity accounted for 24.77% and 15.26% of the total variance, with no distinct separation of bacterial communities across groups (Figure 2E).

At the phylum level, the dominant taxa were *Bacteroidota* (60.20%), *Bacillota* (30.87%), and *Pseudomonadota* (4.10%), with no significant differences between groups (*p* > 0.05, Figure 2F, Table 8). At the genus level, the ruminal microbiome was dominated by *Xylanibacter* (33.40%), *norank_f__F082* (6.77%), *Rikenellaceae_RC9_gut_group* (6.78%), *Christensenellaceae_R-7_group* (3.98%), and *NK4A214_group* (3.73%) (Figure 2G). Compared with the CON group, the RE group had significantly increased relative abundances of *Rikenellaceae_RC9_gut_group* and *Butyrivibrio* (*p* < 0.05, Figure 2H,I). LEfSe analysis identified five bacterial biomarkers (LDA score > 2.5, *p* < 0.05): the RE group was enriched with *Bacillota* members, including *Butyrivibrio*, *norank_f__Lachnospiraceae*, *[Eubacterium]_xylanophilum_group*, and *probable_genus_10* (all belonging to *Lachnospiraceae* family), while the CON group had a higher abundance of *Acetitomaculum* (Figure 2J).

## 4. Discussion

During hot summer months, elevated ambient temperature and humidity create unfavorable conditions for dairy ruminants: metabolic heat cannot be dissipated promptly, leading to increased body temperature and ultimately heat stress [23]. Although the temperature-humidity index is widely used to assess heat stress in cows, it is not applicable to buffaloes. Our laboratory recently developed and validated a thermal comfort assessment model for dairy buffaloes under hot and humid climates, with the model integrating RR and BST showing the highest accuracy in reflecting thermal status [21]. In the current study, this model confirmed that both groups experienced severe and sustained heat stress, with the RE group showing partial relief, a finding consistent with previous reports that RE mitigates heat stress in broiler chickens [24], supporting its broad potential as a heat stress modulator in livestock. Heat stress triggers adaptive thermoregulatory mechanisms, and elevated BST is a direct indicator of excess body heat accumulation when heat production exceeds heat-dissipating capacity [25]. The tendency of RE to reduce BST provides direct evidence of its ability to alleviate thermal discomfort in heat-stressed dairy buffaloes, though it should be noted that this reduction only exhibited a statistical trend rather than a significant difference, which may be attributed to the relatively small sample size in the present study. However, a notable limitation of this study is the use of a single RE supplementation dose, which was referenced to the effective concentrations reported in previous Sanhe dairy cow studies [19]. Without multiple dose gradients, we cannot establish a dose–response relationship or confirm whether this dose is optimal. This information is critical for practical feeding recommendations, as it helps balance efficacy and cost-effectiveness in dairy buffalo farms. Future research should include multiple RE dose levels to identify the optimal inclusion concentration and clarify its safety range for dairy buffaloes.

Heat stress poses a severe challenge to lactating buffaloes: high nutrient demands for milk synthesis increase metabolic heat production, and persistent high temperature/humidity exacerbate heat stress [26]. Recent studies demonstrate that heat stress exerts a detrimental effect on milk protein concentration by inhibiting the synthesis of key proteins (e.g., α-casein, β-casein) and compromises milk nutritional value by lowering phospholipid content [27]. Dietary phytogenic compounds (e.g., bupleurum, honeysuckle extracts) have been shown to mitigate these effects in dairy cows [28,29], and plant extracts can optimize milk fatty acid profiles in dairy ewes by reducing ruminal biohydrogenation of fatty acids [30,31]. In line with these previous findings, our study demonstrated increased milk production, 4% FCM, milk protein, lactose, and SNF concentrations in the RE group. Notably, RE supplementation reduced the proportions of milk SFAs (specifically C14:0 and C18:0) while increasing those of PUFAs (namely C22:5n3 [DPA] and C22:5n6). These observed fatty acid alterations may be indicative of a potential shift toward a more favorable milk fatty acid profile, though any inferences regarding corresponding implications for human nutritional value, such as putative anti-inflammatory and cardiovascular protective effects, still require further validation through targeted human nutrition studies. Importantly, no differences in DMI or nutrient digestibility were observed between groups, ruling out the possibility that RE’s effects on lactation were driven by increased feed intake or improved nutrient absorption. This aligns with Kholif et al. [32], who reported that 10 g/d of whole rosemary plant improved milk yield in dairy goats without affecting feed intake. Collectively, these studies confirm the positive impact of rosemary supplementation on ruminant milk production, regardless of the forms (extract, raw plant, essential oil) or environmental conditions. It is notable that, as a methodological consideration, future studies could adopt linear mixed-effects models to better address the repeated-measure nature of the data (e.g., milk yield), thereby enhancing statistical rigor.

Heat stress induces oxidative stress, which contributes to dysregulated inflammatory responses in ruminants [33]. Recognized as a safe food component by the European Food Safety Authority (EFSA), RE is enriched with polyphenols (carnosic acid, rosmarinic acid, carnosol) that enhance enzymatic (CAT, GSH-PX, SOD) and non-enzymatic (glutathione) antioxidant systems while suppressing systemic inflammation [34]. Carnosic acid, the most abundant and potent antioxidant in RE, retains its efficacy in lambs even after ruminal fermentation [35,36], and intravenous infusion of carnosic acid modulates oxidative stress and inflammatory response in transition dairy cows, with the underlying mechanism linked to the activation of the PI3K/AKT/Nrf2 pathway [37,38]. Beyond direct antioxidant effects, HSP70 acts as a cellular “thermometer” that mediates heat stress signaling, supports thermotolerance, and coordinates oxidative damage sensing and immune function modulation [39]. Tang et al. [23] reported that purified RE supplementation enhances the antioxidant status and upregulates HSP70 expression in the heart of heat-stressed chicken, and our study similarly observed increases in serum CAT activity along with T-AOC and HSP70 concentrations in the RE group. Concomitantly, RE supplementation elevated serum IgA/IgM and reduced IL-1β/TNF-α, confirming improved antioxidant homeostasis and immune function, effects that align with Kong et al. [16], who reported comparable benefits in high-producing dairy cows. A key limitation of this study, however, is the single-time-point blood sampling at the end of the experiment, which prevents tracking the temporal dynamics of these serum indicators.

Plant extracts act as rumen modifiers via selective antimicrobial activity [40], and our study showed that RE enriched several specific functional taxa in the rumen of dairy buffaloes, potentially associated with changes in VFA concentration and composition, rather than inducing a global shift in the rumen microbial community. Higher α-diversity (species richness indices-Sobs, Ace) in the RE group enhances rumen ecosystem resistance to heat stress-induced dysbiosis by providing functional redundancy [41]. This ecological resilience was further supported by the unchanged rumen pH in our RE group, which aligns with previous findings in dairy cows and Damascus goats [16,32]. The VFA are typically regarded as the main carbon source for rumen microbes, accounting for approximately 70% of the digestible energy in ruminants [42]. In the present study, the simultaneous increase in acetate, propionate, butyrate, and TVFA in the RE group reflects improved rumen function and nutrient utilization. Elevated propionate (the primary gluconeogenic precursor) was detected in the RE group, coinciding with higher milk lactose content. This observation supports the plausible hypothesis that increased propionate may facilitate hepatic glucose synthesis, which could, in turn, support mammary lactose synthesis and immune cell function [43]. This finding aligns with the reports of Torres et al. [43] and Kong et al. [16], who documented increased propionate levels in sheep and dairy cows supplemented with essential oils (enriched with carnosic acid and carvacrol) or RE. Higher propionate production also suggests potential methane mitigation by competing with methanogenesis for hydrogen, redirecting metabolic flux to energy-efficient VFA synthesis [44], though direct methane measurements in RE-supplemented dairy buffaloes are needed.

Beyond enhancing overall diversity, RE supplementation specifically enriched functionally critical bacterial genera. *Rikenellaceae_RC9_gut_group* (*Rikenellaceae* family) is known to specialize in structural carbohydrate degradation and producing acetate and propionate [7,45], with its abundance correlating positively with acetate concentration [46]. *Butyrivibrio* spp. (family *Lachnospiraceae*) can degrade both structural and non-structural carbohydrates and synthesizes butyrate (a VFA that promotes rumen epithelial proliferation and suppresses pro-inflammatory signaling [47,48]), hinting at a potential synergistic effect between the metabolism of this genus and RE’s anti-inflammatory properties. Under the co-enrichment of *Rikenellaceae_RC9_gut_group* and *Butyrivibrio* spp., increased production of “acetate + propionate + butyrate” was observed in the RE group. This co-occurrence suggests a plausible scenario where these microbial metabolites may provide sufficient substrates for milk component synthesis and contribute to rumen epithelial health. Future studies should integrate multi-omics analyses (metabolomics, metatranscriptomics) and in vitro validation experiments to confirm these potential mechanistic links. LEfSe analysis further identified that multiple bacterial genera within the family *Lachnospiraceae* were enriched in the RE group. As a core taxonomic group in the rumen microbiota, the *Lachnospiraceae* family plays well-documented roles in carbohydrate metabolism, VFA synthesis, and rumen barrier maintenance [49,50]. The enrichment of these functionally distinct genera in the RE group is associated with enhanced fermentation efficiency, optimized nutrient utilization, and strengthened physiological resilience to heat stress. These observations collectively support the formation of a potential “metabolic network” which may contribute to the stabilization of the rumen ecosystem.

## 5. Conclusions

The supplementation of the diet with 20 g/d of RE was associated with improvements in lactation performance indicators of dairy buffaloes under hot weather by improving antioxidant capacity, regulating immune function, and optimizing ruminal fermentation. These beneficial effects are associated with specific shifts in the ruminal microbial community (e.g., enrichment of *Rikenellaceae_RC9_gut_group* and multiple genera within the *Lachnospiraceaea* family). Taken together, these results underscore RE’s potential as a natural feed supplement to improve the adaptability of dairy buffaloes to hot environmental conditions and boost lactation productivity, providing meaningful practical implications for refining buffalo production systems in tropical and subtropical areas. Further long-term studies with larger sample sizes are required to verify the sustained efficacy of RE supplementation in heat-stressed dairy buffaloes.

## Figures and Tables

**Table 1:** Ingredients and nutritional components of the basal diet (DM basis, %).

Item	Content
Ingredients	
* Pennisetum purpureum* Schumach. hay	12.00
* *Corn silage	29.28
* *Brewer’s grains	40.51
* *Concentrated feed mixture ^(1)^	17.21
* *Premix ^(2)^	0.50
* *NaCl	0.50
* *Total	100.00
Nutrient levels ^(3)^	
* *GE, MJ/kg	17.10
* *CP	18.94
* *CA	7.18
* *NDF	45.16
* *ADF	23.67
* *Ca	1.83
* *P	0.37

GE—gross energy; CA—crude ash; CP—crude protein; NDF—neutral detergent fiber; ADF—acid detergent fiber. ^(1)^ The concentrated feed mixture was sourced from a local livestock and poultry feed company in Nanning, and its nutritional composition (on an as-fed basis) was as follows: DM 91.50%, CP 20.05%, crude ash 7.93%, NDF 15.44%, ADF 6.52%, and GE 19.04 MJ/kg. ^(2)^ Premix provided the following per kg of the diet: VA 550,000 IU, VE 3000 IU, VD 3150,000 IU, Fe (as ferrous sulfate) 4.0 g, Cu (as copper sulfate) 1.3 g, Mn (as manganese sulfate) 3.0 g, Zn (as zinc sulfate) 6.0 g, Co (as Cobalt sulfate) 80.0 mg. ^(3)^ All the nutrient levels are measured values.

**Table 2:** Effects of RE supplementation on physiological indices in *Mediterranean* dairy buffaloes under hot weather.

Item	Treatments		*p*-Value
	CON	RE	
RR, breaths/min	32.10 ± 1.09	29.73 ± 1.24	0.168
BST, °C	35.93 ± 0.04	35.80 ± 0.05	0.073
RT, °C	38.62 ± 0.06	38.67 ± 0.04	0.504

**Table 3:** Effects of RE supplementation on the dry matter intake and nutrient apparent digestibility in *Mediterranean* dairy buffaloes under hot weather.

Item	Treatments		*p*-Value
	CON	RE	
DMI, kg/d	15.03 ± 0.30	15.09 ± 0.43	0.919
CP, %	58.56 ± 1.84	59.74 ± 1.48	0.621
NDF, %	58.11 ± 1.05	58.16 ± 1.62	0.976
ADF, %	33.66 ± 1.35	35.92 ± 1.79	0.335
EE, %	60.98 ± 2.48	59.04 ± 2.83	0.617

**Table 4:** Effects of RE supplementation on milk traits in *Mediterranean* dairy buffaloes under hot weather.

Item	Treatments		*p*-Value
	CON	RE	
Milk production, kg/d	6.76 ± 0.03 ^b^	7.12 ± 0.05 ^a^	<0.001
4%FCM ^1^, kg/d	10.66 ± 0.05 ^b^	11.40 ± 0.08 ^a^	<0.001
Milk fat, %	7.85 ± 0.46	8.02 ± 0.35	0.782
Milk protein, %	4.59 ± 0.02 ^b^	4.84 ± 0.04 ^a^	<0.001
Milk lactose, %	5.01 ± 0.02 ^b^	5.08 ± 0.03 ^a^	0.032
SNF, %	10.26 ± 0.03 ^b^	10.41 ± 0.03 ^a^	<0.001
TS, %	18.34 ± 0.50	18.47 ± 0.36	0.844
MUN, mg/dL	21.80 ± 0.88	22.44 ± 0.73	0.583
FFA, mmol/L	0.41 ± 0.01	0.44 ± 0.01	0.057

^a,b^ Mean values within a row with different superscripts statistically differed at *p* < 0.05. ^1^ 4%FCM = 0.4 × milk production (kg/d) + 0.15 × milk fat (%) × milk production (kg/d).

**Table 5:** Effects of RE supplementation on the main milk fatty acid profile in *Mediterranean* dairy buffaloes under hot weather.

Item	Treatments		*p*-Value
	CON	RE	
C10:0, μg/mL	313.32 ± 28.25	304.03 ± 17.10	0.782
C12:0, μg/mL	602.12 ± 44.10	593.45 ± 29.96	0.872
C14:0, μg/mL	304.46 ± 25.82 ^a^	196.25 ± 23.31 ^b^	<0.001
C15:0, μg/mL	217.75 ± 15.89	211.47 ± 23.05	0.825
C16:0, μg/mL	352.73 ± 41.30	358.10 ± 15.47	0.905
C18:2 (9-CIS, 11-TRANS), μg/mL	209.18 ± 22.78	238.37 ± 32.75	0.474
C18:2(10-TRANS, 12-CIS), μg/mL	411.54 ± 25.98	468.97 ± 34.20	0.198
C18:1n9c, μg/mL	280.23 ± 47.01	281.53 ± 43.30	0.984
C18:1n9t, μg/mL	379.41 ± 48.63	349.89 ± 46.22	0.665
C18:0, μg/mL	331.22 ± 24.91 ^a^	167.14 ± 32.49 ^b^	<0.001
C20:5n3 (EPA), μg/mL	332.66 ± 30.20	317.45 ± 10.64	0.641
C20:3n3, μg/mL	8.97 ± 1.29 ^b^	13.85 ± 0.80 ^a^	<0.001
C20:1, μg/mL	31.86 ± 5.44 ^b^	61.24 ± 4.04 ^a^	<0.001
C21:0, μg/mL	358.07 ± 24.92	390.67 ± 15.51	0.281
C22:5n3 (DPA), μg/mL	14.08 ± 1.25 ^b^	20.15 ± 1.79 ^a^	0.012
C22:5n6, μg/mL	32.13 ± 3.04 ^b^	40.95 ± 2.76 ^a^	0.046
C22:1n9, μg/mL	25.65 ± 2.98	34.51 ± 3.59	0.074
SFA, μg/mL	3199.52 ± 141.26	2916.61 ± 82.70	0.085
UFA, μg/mL	2869.63 ± 99.43	3065.76 ± 134.43	0.268
SFA/UFA	1.12 ± 0.05	0.97 ± 0.05	0.061
MUFA, μg/mL	1192.31 ± 69.41	1254.18 ± 87.76	0.587
PUFA, μg/mL	1677.32 ± 54.80	1806.57 ± 68.71	0.095
CLA ^1^, μg/mL	620.72 ± 43.43	707.34 ± 54.44	0.229

^a,b^ Mean values within a row with different superscripts statistically differed at *p* < 0.05. ^1^ CLA included the C18:2 (9-CIS, 11-TRANS) and C18:2 (10-TRANS, 12-CIS) fatty acids.

**Table 6:** Effects of RE supplementation on blood biochemical, hormonal, antioxidative, and immune indices in *Mediterranean* dairy buffaloes under hot weather.

Item	Treatments		*p*-Value
	CON	RE	
Biochemical indices			
TP, g/L	72.16 ± 1.79	74.35 ± 1.40	0.343
ALB, g/L	37.03 ± 0.93	37.35 ± 1.00	0.821
GLO, g/L	35.12 ± 1.55	36.87 ± 1.61	0.446
A: G	1.09 ± 0.05	1.03 ± 0.06	0.482
ALP, U/L	173.44 ± 16.75	219.33 ± 14.59	0.055
CK, U/L	168.78 ± 13.21	161.89 ± 14.78	0.733
LDH, U/L	596.88 ± 19.31	528.75 ± 21.88	0.636
AST, U/L	137.00 ± 5.66	145.90 ± 9.75	0.454
ALT, U/L	52.38 ± 2.51	50.80 ± 2.29	0.650
AST: ALT	2.56 ± 0.14	2.89 ± 0.16	0.151
UN, mmol/L	6.21 ± 0.28	6.42 ± 0.23	0.570
GLU, mmol/L	2.32 ± 0.22	2.78 ± 0.12	0.117
TG, mmol/L	0.23 ± 0.04	0.25 ± 0.03	0.743
TC, mmol/L	2.98 ± 0.10	2.66 ± 0.13	0.075
Hormone levels			
T_3_, ng/mL	1.14 ± 0.04	1.02 ± 0.06	0.128
T_4_, ug/dL	4.24 ± 0.28	3.65 ± 0.14	0.067
INS, uIU/mL	7.01 ± 0.70	6.92 ± 0.77	0.933
Oxidative status			
CAT, U/mL	1.80 ± 0.21 ^b^	3.92 ± 0.46 ^a^	<0.001
GSH-PX, U/mL	229.10 ± 12.60	258.01 ± 31.17	0.409
SOD, U/mL	23.01 ± 2.57	25.92 ± 1.12	0.764
T-AOC, nmol/mL	799.40 ± 20.43 ^b^	880.75 ± 22.66 ^a^	0.017
MDA, nmol/mL	1.75 ± 0.21	1.68 ± 0.16	0.771
Immune indices			
IgA, mg/dL	0.044 ± 0.002 ^b^	0.054 ± 0.003 ^a^	0.039
IgG, mg/dL	0.088 ± 0.003	0.083 ± 0.005	0.318
IgM, mg/dL	0.50 ± 0.05 ^b^	0.66 ± 0.05 ^a^	0.029
HSP70, pg/mL	35.92 ± 1.93	39.98 ± 1.04	0.073
SAA, μg/mL	2.63 ± 0.15	2.47 ± 0.15	0.462
IL-1β, pg/mL	143.32 ± 4.28 ^a^	120.04 ± 5.35 ^b^	<0.001
IL-2, pg/mL	56.42 ± 6.31	53.30 ± 3.46	0.672
IL-6, pg/mL	167.62 ± 8.98	152.46 ± 5.40	0.170
TNF-α, pg/mL	17.69 ± 0.51 ^a^	14.12 ± 0.83 ^b^	<0.001
IFN-γ, pg/mL	347.75 ± 20.65	321.62 ± 17.01	0.339

^a,b^ Mean values within a row with different superscripts statistically differed at *p* < 0.05.

**Table 7:** Effects of RE supplementation on rumen fermentation profile in *Mediterranean* dairy buffaloes under hot weather.

Item	Treatments		*p*-Value
	CON	RE	
pH	6.76 ± 0.10	6.85 ± 0.09	0.536
MCP, mg/dL	12.25 ± 0.33	12.71 ± 1.06	0.694
NH_3_-N, mg/dL	8.71 ± 0.44	8.28 ± 0.57	0.582
Acetate, mmol/L	56.86 ± 2.15 ^b^	75.96 ± 2.05 ^a^	<0.001
Propionate, mmol/L	14.55 ± 0.51 ^b^	21.10 ± 0.53 ^a^	<0.001
A:P	3.95 ± 0.26	3.60 ± 0.06	0.248
Butyrate, mmol/L	8.95 ± 0.44 ^b^	13.21 ± 0.68 ^a^	<0.001
Isobutyrate, mmol/L	1.21 ± 0.07	1.31 ± 0.07	0.385
Valerate, mmol/L	1.25 ± 0.03	1.31 ± 0.09	0.532
Isovalerate, mmol/L	1.49 ± 0.09	1.61 ± 0.07	0.361
TVFA, mmol/L	84.32 ± 1.46 ^b^	114.48 ± 2.83 ^a^	<0.001

^a,b^ Mean values within a row with different superscripts statistically differed at *p* < 0.05.

**Table 8:** Effects of RE supplementation on ruminal bacterial abundance at the phylum level (%) in *Mediterranean* dairy buffaloes under hot weather.

Item	Treatments		*p*-Value
	CON	RE	
*Bacteroidota*	60.37 ± 4.53	60.03 ± 2.78	0.952
*Bacillota*	30.84 ± 4.10	30.89 ± 1.68	0.992
*Pseudomonadota*	3.64 ± 0.97	4.56 ± 1.13	0.559
*Spirochaetota*	1.34 ± 0.38	1.08 ± 0.21	0.569
*Fibrobacterota*	0.95 ± 0.25	1.18 ± 0.22	0.513
*Patescibacteria*	0.96 ± 0.21	0.79 ± 0.21	0.587
*Cyanobacteriota*	0.66 ± 0.15	0.50 ± 0.15	0.482
*Verrucomicrobiota*	0.56 ± 0.19	0.35 ± 0.10	0.378
*Thermodesulfobacteriota*	0.15 ± 0.04	0.18 ± 0.02	0.576
*unclassified_k__norank_d__Bacteria*	0.14 ± 0.02	0.11 ± 0.04	0.585

## Data Availability

The data used to support the findings of this study are available from the corresponding author upon request.

## Supplementary Materials

The following supporting information can be downloaded at: https://www.mdpi.com/article/10.3390/ani16020216/s1, Table S1: Effects of RE supplementation on the minor milk fatty acid profile in *Mediterranean* dairy buffaloes under hot weather.

## Abbreviations

The following abbreviations are used in this manuscript:

4% FCM	4% fat-corrected milk
ADF	Acid detergent fiber
ALB	Albumin
ALP	Alkaline phosphatase
ALT	Alanine transaminase
AST	Aspartate aminotransferase
BST	Body surface temperature
CAT	Catalase
CK	Creatine kinase
CP	Crude protein
DM	Dry matter
EE	Ether extract
FFA	Free fatty acids
GLU	Glucose
GSH-PX	Glutathione peroxidase
HSP70	Heat shock protein 70
IFN-γ	Interferon-γ
IgG	Immunoglobulin G
IL-1β	Interleukin-1beta
INS	Insulin
LDA	Linear discriminant analysis
LDH	Lactate dehydrogenase
MCP	Microbial crude protein
MDA	Malondialdehyde
MUFA	Monounsaturated fatty acid
MUN	Milk urea nitrogen
NH3-N	Ammonia nitrogen
P3	Physiological index
PCoA	Principal coordinate analysis
PUFA	Polyunsaturated fatty acid
RE	Rosemary extract
RR	Respiratory rate
RT	Rectal temperature
SAA	Serum amyloid A
SFA	Saturated fatty acid
SNF	Solids-not-fat
SOD	Superoxide dismutase
T3	Triiodothyronine
T4	Tetraiodothyronine
T-AOC	Total antioxidant capacity
TC	Total cholesterol
TG	Triglyceride
THI	Temperature–humidity index
TMR	Total mixed ration
TNF-α	Tumor necrosis factor-α
TP	Total protein
TS	Total solids
UFA	Unsaturated fatty acid
UN	Urea nitrogen
VFA	Volatile fatty acid
