# Supplementary material for: Supplementation of Rosemary Extract Improves Lactation Performance and Rumen Function in Dairy Buffaloes Under Hot Weather
Source: Animals (Basel). 2026 Jan 11;16(2):216. doi: 10.3390/ani16020216 (PMC12838054; doi:10.3390/ani16020216)
Supplement: Supplementary file 1 [file animals-16-00216-s001.zip › animals-4067690-supplementary.pdf]

**Table S1.** Effects of RE supplementation on the minor milk fatty acid profile in *Mediterranean* dairy buffaloes under hot weather.

| Item | Treatments | | *p*-value |
|---|---|---|---|
| | CON | RE | |
| C8:0, μg/mL | 13.65±2.04 | 17.28±1.40 | 0.160 |
| C11:0, μg/mL | 8.62±1.75 | 7.08±1.24 | 0.480 |
| C13:0, μg/mL | 56.08±7.99 | 54.25±5.28 | 0.850 |
| C14:1n5, μg/mL | 24.46±3.67 | 28.39±3.15 | 0.427 |
| C15:1n5, μg/mL | 148.97±5.56 | 148.82±9.95 | 0.990 |
| C16:1n7, μg/mL | 262.56±27.15 | 309.59±16.47 | 0.160 |
| C17:1n7, μg/mL | 6.69±0.97 | 7.96±1.22 | 0.427 |
| C17:0, μg/mL | 185.33±21.67 | 201.21±12.07 | 0.532 |
| C18:3n6, μg/mL | 140.62±21.09 | 163.21±15.48 | 0.399 |
| C18:3n3, μg/mL | 155.86±12.15 | 153.99±16.59 | 0.929 |
| C18:2n6c, μg/mL | 17.06±2.27 | 16.43±1.40 | 0.817 |
| C20:4n6 (ARA), μg/mL | 11.11±2.19 | 10.89±0.95 | 0.928 |
| C20:3n6, μg/mL | 92.46±9.34 | 110.04±10.79 | 0.234 |
| C20:2, μg/mL | 198.61±17.17 | 209.10±16.16 | 0.662 |
| C20:0, μg/mL | 84.64±7.81 | 82.03±12.33 | 0.860 |
| C22:6n3 (DHA), μg/mL | 53.05±5.13 | 43.16±3.10 | 0.116 |
| C22:1T, μg/mL | 16.94±1.26 | 16.80±1.14 | 0.938 |
| C22:0, μg/mL | 165.36±14.03 | 150.76±7.75 | 0.378 |
| C23:0, μg/mL | 97.28±8.68 | 85.39±5.42 | 0.260 |
| C24:1n9, μg/mL | 15.54±1.44 | 15.45±1.19 | 0.962 |
| C24:0, μg/mL | 108.88±9.53 | 97.51±6.08 | 0.328 |
